# Mechanism of Circadian Regulation in Ferroptosis of the BMAL1/NRF2 Pathway in Renal Ischemia–Reperfusion

**DOI:** 10.3390/biomedicines13061375

**Published:** 2025-06-04

**Authors:** Shang Xu, Qiao Tang, Haiyang Du, Jiatao Xie, Ruoxin He, Ruiyan Wang, Qian Sun

**Affiliations:** Department of Anesthesiology, Renmin Hospital of Wuhan University, No. 238 Jiefang Road, Wuchang District, Wuhan 430061, China; 2021305231022@whu.edu.cn (S.X.); tangqiao20212021@163.com (Q.T.); 2021305231069@whu.edu.cn (H.D.); 2022305232033@whu.edu.cn (J.X.); 2024305232152@whu.edu.cn (R.H.); 2023305233118@whu.edu.cn (R.W.)

**Keywords:** ferroptosis, renal ischemia–reperfusion injury, circadian rhythm, *BMAL1*, *NRF2*

## Abstract

**Background**: Renal ischemia–reperfusion injury (IRI) is a frequent cause of kidney transplant failure. Recent studies have shown that the extent of injury is closely linked to ferroptosis, and the process of cellular ferroptosis is diurnal and regulated by circadian genes. *NRF2*, involved in iron–heme metabolism, may be related to ferroptosis. We hypothesize that the pathway plays a role in circadian regulation in ferroptosis in renal IRI. **Methods**: Using hematoxylin and eosin (H&E) staining, enzyme-linked immunosorbent assay (ELISA), Cell Counting Kit-8 (CCK8), flow cytometry, real-time quantitative reverse transcription PCR (qRT-PCR), and Western blotting, we analyzed renal tubular tissues in vivo and in vitro and compared the groups with IR injury treatment, inhibition of ferroptosis, and inhibition of *BMAL1* gene expression at the ZT0 (zeitgeber time 0) and ZT12 (zeitgeber time 12) time points. **Results**: IR injury treatments caused exacerbation of injury, both in vivo and in vitro, and were more pronounced at the ZT12 time point, which correlates with circadian rhythms. The use of the ferroptosis inhibitor (Fer-I) attenuated IR injury, suggesting that IRI is associated with ferroptosis. In contrast, reduced *BMAL1*-gene expression exacerbated injury, and *NRF2*, which is elevated in IR injury, was suppressed. **Conclusions**: The circadian gene *BMAL1* affects the circadian rhythm of ferroptosis in renal IRI through the regulation of *NRF2* and its downstream pathway. In this study, renal injury is well ameliorated by the ferroptosis inhibitor, exhibiting potential as a therapeutic agent for use in renal transplantation.

## 1. Introduction

Renal IRI is a common clinical form of kidney injury. Renal IRI can occur secondary to kidney transplantation and under a variety of other conditions. Studies have shown that IRI and its subsequent complications are independent risk factors affecting the long-term survival of the transplanted kidney [1]. It is noteworthy that in recent years, ferroptosis, as a novel mode of programmed cell death, has received attention for its role in acute kidney injury and renal IRI. During IRI, renal tissue undergoes a transition from hypoxia to reoxygenation, which is accompanied by increased oxidative stress and inflammatory responses. It has been shown that the extent of renal injury after IRI is closely related to the onset of ferroptosis [2]. The overloading of intracellular iron ions and the accumulation of reactive oxygen species leads to the eventual movement of the cell membrane towards lipid peroxidation and cell death.

Both in vivo and in vitro, the oscillator plays an important role in circadian rhythm. It consists of a group of recently expressed genes (*CLOCK*, *BMAL1*, *PER*, *CRY*, *DEC*, etc.) and their encoded proteins (CLOCK, BMAL1, PER, CRY, DEC, etc.). In this oscillator, the heterodimeric transcription factor *CLOCK/BMAL1* drives the expression of the PER and CRY proteins, which are self-repressors of *PER* and *CRY*. Reactivation of *CLOCK-BMAL1* occurs a few hours after the renewal of the PER and CRY proteins [3], and tissue-specific rhythmic oscillatory synchronization factors are stored at the cellular and tissue levels for the maintenance of circadian rhythms in vitro [4]. Previous studies have also shown that circadian gene expression in mammalian tissue culture cells can be induced by a serum shock [5].

Circadian rhythms adapt to daily environmental changes by regulating many physiological pathways, and both oxidative stress injury and cellular ferroptosis in IRI are markedly circadian in nature. There is daily variability in oxidative stress metrics in models of kidney injury at different time points within a day, and the disruption of circadian rhythm genes can exacerbate oxidative stress, thereby aggravating kidney injury [6]. Studies have shown that the pathophysiological process of cellular ferroptosis also exhibits circadian variability and is regulated by circadian genes [7]. The *BMAL1* gene plays a central regulatory role in circadian variability, and *NRF2* is a core transcription factor for anti-oxidative stress. *BMAL1* binds to the PPAR promoter through the *E-box* element and regulates the expression of *NRF2* and its downstream anti-oxidative stress proteins, where the circadian rhythm of the *NRF2/ARE* pathway is critical for the regulation of anti-oxidative stress in a renal IRI model [6]. *NRF2* is associated with the homeostasis of iron–heme metabolism, and aberrant *NRF2* signaling may lead to an increase in ferroptosis [7]. The *BMAL1/NRF2* pathway is not only involved in the regulation of oxidative stress [8], but may also influence ferroptosis through the modulation of intracellular iron metabolism and antioxidant enzyme activities.

Therefore, exploring ferroptosis and its circadian rhythm changes in the renal IRI model is crucial for a deeper understanding of the pathological mechanisms of renal IRI and the development of new therapeutic strategies. In this paper, we explored the circadian rhythm of ferroptosis and its underlying mechanisms in the renal IRI model, providing new insights for the perioperative prevention of long-term graft dysfunction. This study also offers potential targets for the development of effective drugs or measures to protect transplant kidneys and reduce IRI, thereby improving patient survival and reducing healthcare costs.

## 2. Materials and Methods

### 2.1. Animal Experiment

#### 2.1.1. Animal Handling Methods

Adult male normal wild-type *C57BL/6* mice (9–12 w, 20–25 g) were obtained from the Wuhan University Laboratory Animal Center, and the experimental protocol was implemented after review and approval by the Experimental Animal Welfare Ethics Review Committee of Wuhan University People’s Hospital (WDR Motion (FU) No. 20210124). The mice were housed under the standard conditions of 20 ± 2 °C, 40% ± 5% relative humidity, and a 12 h light–dark cycle. ZT0 and ZT12 were used as time references, with ZT0 (zeitgeber time 0) representing the beginning of the light period and ZT12 representing the beginning of the night or dark period. Forty-eight adult male C57BL/6 mice were randomly divided into the sham operation group (S group), the renal ischemia–reperfusion group (IR group), the ferroptosis inhibitor group (IR+ Fer-I group), and the *BMAL1* knockdown group (IR+*BMAL1-* group) at the ZT0 and ZT12 time points, respectively (n = 6). Anesthesia was induced with 50 mg/kg amobarbital, the abdominal cavity was opened to clamp the bilateral renal clitoris for 30 min, and then after 24 h of reperfusion to establish the IR model, the mice were euthanized, and kidney and blood samples were collected for subsequent analysis. The S group also underwent the surgery, but the bilateral renal clitoris was not clamped. The ferroptosis inhibitor group was injected intraperitoneally with Ferrostatin-1 (Fer-I, cas-no347174-05-4, 10 mg/kg) three times; the first dose was administered 1 h before modeling, and the additional does were given every 8 h. The *BMAL1* knockdown group was injected with *BMAL1* shRNA scAAV9 (Heyuan Biotechnology Co., Ltd., Shanghai, China), 0.125 mL/pc, with a viral titer of 2.5 × 10^12^ vg/mL, by tail vein 2 weeks before modeling.

#### 2.1.2. Renal Function Assay

Blood samples were centrifuged at 4500 rpm for 10 min at 4 °C, and plasma was collected and stored at −20 °C. BUN (blood urea nitrogen) and CREA (creatinine) levels were detected by an automatic biochemical analyzer [9].

#### 2.1.3. Renal Histopathology

Rows of longitudinal axis coronal plane-cut kidney tissues were placed in 10% neutral formaldehyde solution, fixe and paraffin embedded, resulting in 4 μm sections, which were routinely stained with hematoxylin and eosin, and observed under an optical microscope to examine the necrosis of the renal tubules. Each mouse kidney section was randomly selected for observation in 20 fields of view, according to the degree of tubular necrosis, scored by Rabb et al.’s semi-quantitative pathology assessment method [10]; the higher the score, the more severe the tubular necrosis (maximum—4 points), with 0 points: normal kidney; 1 point: minimal necrosis (<5% of tubular necrosis); 2 points: mild necrosis (5–25% of tubular necrosis); 3 points: moderate necrosis (25–75% tubular necrosis); 4 points: severe necrosis (>75% tubular necrosis).

#### 2.1.4. Determination of IL-1β, IL-6, IL-10, and TNF-β Levels in Plasma

Plasma levels of IL-1β, IL-6, IL-10, and TNF-β were measured using enzyme-linked immunosorbent assay (ELASA), and the calculated results were expressed as the optical density at 450 nm. The ELISA kits were purchased from Elabscience (Wuhan, China), and were employed according to the manufacturer’s instructions. (Mouse IL-1β ELISA Kit, Elabscience, E-EL-M0037; Mouse IL-6 ELISA Kitt, Elabscience, E-EL-M0044; Mouse IL-10 ELISA Kit, Elabscience, E-EL-M0046; Mouse TNF-β ELISA Kit, Elabscience, E-EL-M1210).

#### 2.1.5. Detection of Total Iron Content, Malondialdehyde (MDA), and Glutathione (GSH)

A tissue iron assay kit (A039-2-1, Nanjing Jiancheng Bioengineering Institute, Nanjing, China) was applied for the iron content assay. According to this scheme, kidney tissue was homogenized in phosphate buffered saline, and then an iron chromogenic agent was added. After vortexing, the mixture was clarified by centrifugation at 1500× *g* for 10 min. Finally, the absorbance was measured at a wavelength of 520 nm. Similar to iron content assay, MDA (A003-1-1) and GSH (A006-2-1) assay kits were both obtained from the Nanjing Jiancheng Bioengineering Institute and were employed according to the manufacturer’s instructions. The results were normalized by protein content.

### 2.2. Cell Experiments

#### 2.2.1. Cell Culture and Transfection

Mouse renal tubular epithelial cells, TCMK-1 (from iCell, iCell-m089, 2024010402), were inoculated in DMEM medium containing 10% FBS and 1% P/S and were cultured at 37 °C in a 5% CO_2_ environment. The cells used in the experiment were in the logarithmic growth phase [11]. The cells were inoculated in 6-well cell culture plates with a density of about 1 × 10^5^−1 × 10^6^ and cultured at 37 °C in a 5% CO_2_ incubator for 24 h to achieve a cell fusion rate of 70–80%; liposomes and *BMAL1*siRNA were inoculated in serum-free DMEM medium and mixed to form a liposome mixture medium, which was added to the cell culture plates and cultured at 37 °C under a 5% CO_2_ environment. After 5 h, the medium was aspirated, 2 mL of DMEM medium containing 5% serum was added, and the cells were incubated at 37 °C, 5% CO_2_, for 48 h.

#### 2.2.2. Rhythmic Induction of Cells [12]

When the cell spreads to 90% of the bottom of the bottle, trypsin digestion is induced, and the cell suspension is obtained, which is then equally inoculated into 7 cell culture dishes, with about 10^6^ cells in each dish. When the cells are attached to the wall and accounted for about 70% of the bottom of the dish, the serum-free medium is changed; after starvation treatment for 24 h, the cells res treated with 50% horse serum medium for 2 h to induce the expression of the cellular rhythmic genes, and then the medium is changed to a serum-free medium for continued cultivation.

#### 2.2.3. Establishment and Grouping of Hypoxia–Reoxygenation (HR) Cell Model

The hypoxia–reoxygenation model was prepared at ZT0 and ZT12: cells were switched to serum-free culture medium and placed in a closed container at 37 °C, 5% CO_2_, 1% O_2_, and 94% N_2_ for 2 h of hypoxia treatment. The cells were then switched to a normal culture medium and placed in an incubator with 5% CO_2_, 21% O_2_, and 74% N_2_ for 2 h of reoxygenation treatment [11]. The cells were divided into the following groups: ① the control group (control group); ② the hypoxia–reoxygenation group (HR group); ③ the hypoxia–reoxygenation group + ferroptosis inhibitor group (HR+ Fer-I group), in which 1 μM Fer-I [13,14] was given at the time of hypoxia, which continued for 2 h, and reoxygenation, which lasted for 2 h, which continued for 24 h; ④ the hypoxia–reoxygenation + *BMAL1* gene silencing cells group (HR+si*BMAL1* group).

#### 2.2.4. Cell Activity Assay

The grouped treated cells were inoculated into a cell culture plate, CCK8 solution was added, and the sample was incubated in 5% CO_2_ at 37 °C for 3 h; the absorbance value (OD 450) of each well was measured by using an enzyme marker.

#### 2.2.5. ROS (Reactive Oxygen Species) Detection

An ROS Assay kit (red fluorescence, from Solarbio, Beijing, China CA1420) was employed for ROS assay. The cells in each group were centrifuged, and the supernatant was discarded; the cell precipitate was then resuspended in an appropriate amount of diluted DHE. After incubation for 20–30 min, the cells were washed with PBS to remove the unbound probes. Next, the fluorescent signal of the ROS was detected, and the amount of ROS was analyzed using fluorescence microscopy or flow cytometry.

#### 2.2.6. Flow Cytometry Apoptosis

The cells were collected after the centrifugation of each group. They were then washed twice with pre-cooled PBS, and then the cells were resuspended by adding a binding buffer. Annexin V-Alexa Fluor 647 and PI were then added, mixing well, and the samples were protected from light and left to react at room temperature for 15 min. A binding buffer was then added, the sample was mixed well, placed on ice, and the samples were assessed by flow cytometry (Beckman Coulter, Pasadena, CA, USA, CytoFLEX S) within 1 h.

#### 2.2.7. qRT-PCR

Total RNA was extracted with TRIzol reagent (Takara, Kyoto, Japan), according to the manufacturer’s instructions, and quantified with a NanoDrop 8000 (Thermo Fisher Scientific, Waltham, MA, USA). Reverse transcription reagent (Tiangen, Beijing, China) was used to synthesize cDNA at 2 μg RNA in a 20 μL reaction system. mRNA levels were detected via qRT-PCR using SYBR Green PCR mix (Vazyme, Nanjing, China) from LightCycler96 (Roche, Basel, Switzerland). The mRNA levels of *TFRC*, *PTGS2*, *GPX4*, and *AXSL4* in the samples were detected by qRT-PCR.

#### 2.2.8. Western Blotting [15]

Equal amounts of proteins (50 µg) were separated by 12% SDS-PAGE at 100 V for 3 h. After electrophoresis, the proteins were transferred to polyvinylidene difluoride membranes and incubated at 200 mA for 70 min. The membranes were incubated with primary antibodies (anti-BMAL1 (1:1000), NRF2 (1:2000), HO-1 (1:3000), and β-Actin protein (1:1000)). After three washes in PBST, the membranes were incubated with horseradish peroxidase-conjugated anti-rabbit immunoglobulin G for 2 h in PBST containing 5% skim milk at a dilution of 1:2000. Immunoreactive bands were visualized via enhanced chemiluminescence (PerkinElmer, Inc., Waltham, MA, USA) and captured on X-ray film. Blots were stained with anti-β-actin antibody, and protein levels were normalized relative to β-actin band density.

### 2.3. Statistical Analysis

Data are presented as the means ± standard deviation (SD). GraphPad Prism version 10.1.0 was employed for statistical analysis, and *p* < 0.05 was considered statistically significant. A Student’s *t*-test was used to compare with two groups, and ANOVA, followed by Tukey’s test, was employed for multiple-group comparisons. The Kruskal–Wallis test was applied to analyze data that did not follow a normal distribution. All experiments and assays were independently repeated at least three times.

## 3. Results

### 3.1. Ferroptosis-Related Factors in Renal IR Exhibit a Circadian Rhythm

**Figure 1:**
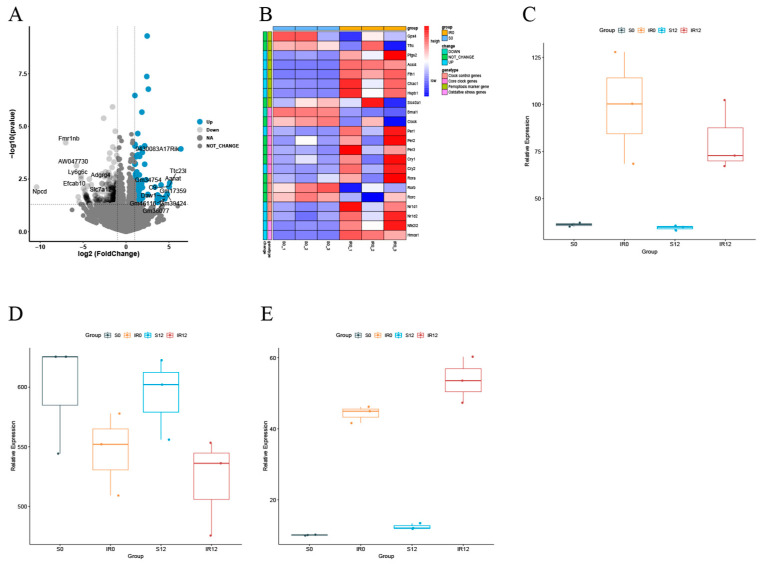
Sequencing analysis results of circadian rhythm-related, oxidative stress-related, and ferroptosis-related genes at ZT0 and ZT12 time points in renal tissues with or without IR. (**A**) Volcano and heat maps of differentially expressed genes in the S and IR groups at the ZT0 time point. (**B**) Volcano and heat maps of differentially expressed genes at the ZT0 and ZT12 time points in the IR group. (**C**) Box plot of *NRF2* gene expression level. (**D**) Box plot of *GPX4* gene expression level. (**E**) Box plot of *ACSL4* gene expression level. Data are expressed as means ± standard deviation (SD).

Based on earlier findings, histological damage was significantly worse in the IR group at ZT12 compared to ZT0, and *BMAL1* could mediate the *NRF2/ARE* pathway to influence tissue damage [16]. Currently, in further differentially expressed gene analysis of renal tissues, with or without IR at the ZT0 and ZT12 time points (Figure 1A,B), it was observed that *BMAL1* was also lower in the IR group at ZT12 compared to ZT0 (*p* < 0.05). Regardless of the ZT0 or ZT12 time points, the *NRF2* gene expression level was higher in the IR group than in the S group, whereas the expression was lower in the IR group at ZT12 compared to ZT0 (Figure 1C). *GPX4*, a crucial gene in the anti-ferroptosis defense system, was identified as displaying lower gene expression levels in the IR group than in the S group at both the ZT0 and ZT12 time points, as well as reduced expression in the IR group at ZT12 compared to ZT0 (Figure 1D). *ACSL4*, a key predictor of susceptibility to ferroptosis, was found to be expressed at a higher level in the IR group than in the S group at both the ZT0 and ZT12 time points, and expression was increased in the IR group at ZT12 compared to ZT0 (Figure 1E). Collectively, these results indicate that IR disrupts the circadian rhythm of ferroptosis-related factors. The involvement of the *BMAL1/NRF2* axis in IR-induced ferroptosis is questionable, deserving further exploration.

### 3.2. Diurnal Changes in IRI and Levels of Ferroptosis-Related Factors

**Figure 2:**
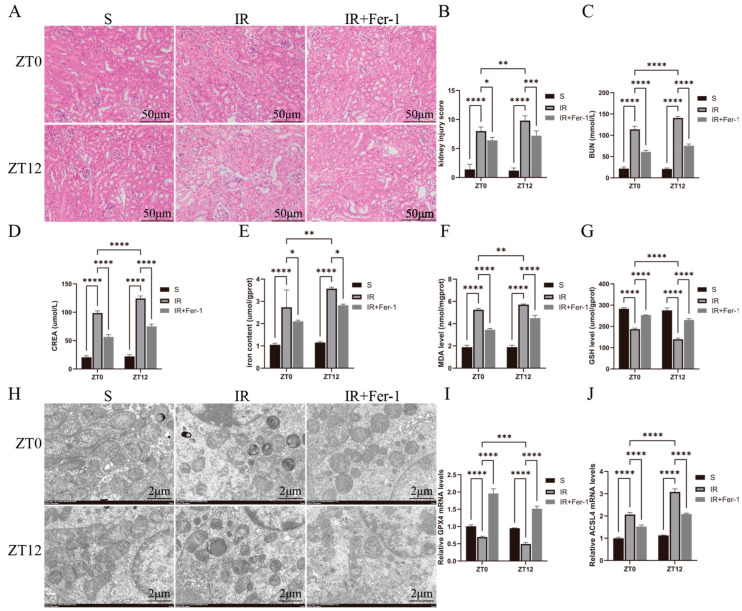
Diurnal changes in renal tissue injury and its ferroptosis-related factor levels. (**A**) Representative H&E-stained images of the kidney used to assess IRI. Scale bar is 50 μm. (**B**) Renal H&E staining for renal injury scoring. (**C**,**D**) BUN and CREA levels in renal IR mice at ZT0 and ZT12 time points. (**E**) Representative images obtained by transmission electron microscopy. Scale bar is 2 μm. (**F**–**H**) Total iron, malondialdehyde, and glutathione levels in renal tissues. (**I**,**J**) RT-qPCR detection of relative expression levels of renal *GPX4* and *ACSL4* mRNA. The mRNA levels in the sham-operated group were normalized to 1. Data are expressed as mean ± standard deviation (SD); * *p* < 0.05; ** *p* < 0.01; *** *p* < 0.001; **** *p* < 0.0001.

In Figure 2A, IR induced renal tubular histological changes, including tubular lumen dilatation, tubular epithelial edema, and vacuolization, are shown. At the ZT0 and ZT12 time points, the histologic damage scores were higher in the IR group than in the S group, and the damage scores were significantly higher at ZT12 than at ZT0 (Figure 2B). Similarly, BUN and CREA levels were higher in the IR group than in the S group at both the ZT0 and ZT12 time points and were further elevated at the ZT12 time point compared with the results at the ZT0 time point (Figure 2C,D). At both the ZT0 and ZT12 time points, the total iron content and MDA levels were significantly higher in the IR group than those in the S group and were further elevated at ZT12 compared with ZT0 (Figure 2E,F). At both the ZT0 and ZT12 time points, the GSH levels and the *GPX4* mRNA levels were significantly reduced in the IR group compared with those in the S group, and both were further reduced at ZT12 compared with the results at ZT0 (Figure 2G,I). Figure 2J shows that the changes in the *ACSL4* mRNA levels were completely opposite to those of *GPX4*. Ferroptosis inhibitor (Fer-I) treatment attenuated IR-induced renal tissue injury, oxidative stress, and lipid peroxidation. Transmission electron microscopy showed that IR induced different degrees of mitochondrial structural disruption in renal cells, especially at the ZT12 time point, which was manifested by rupture of the outer mitochondrial membrane and the swelling, disintegration, and even disappearance of the mitochondrial cristae (Figure 2H). In summary, nocturnal IR led to more severe tissue and functional damage in renal tissues, which may be related to the diurnal variability of cellular ferroptosis.

### 3.3. Diurnal Changes in TCMK-1 Cell Activity, ROS, Ferroptosis-Related Factor, and Inflammatory Factor Levels

**Figure 3:**
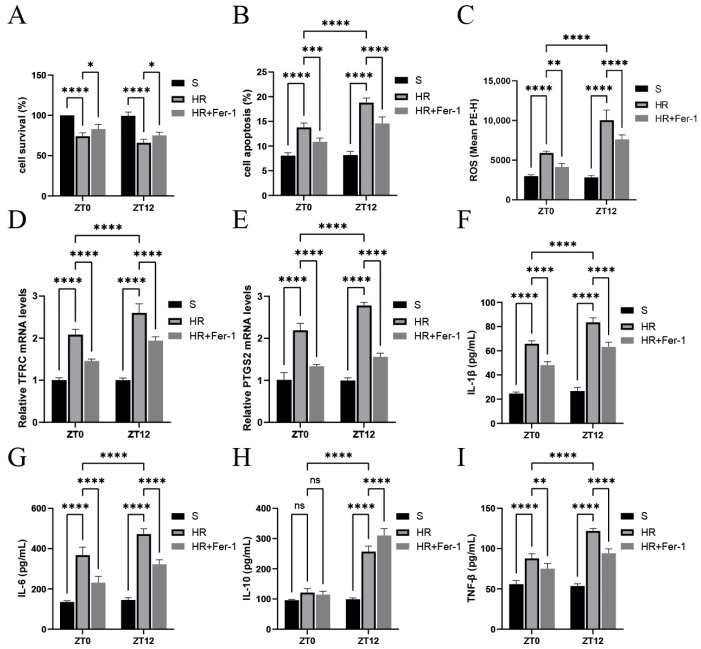
Diurnal changes in renal tubular epithelial cell activity, ROS, ferroptosis-related factors, and inflammatory factor levels. (**A**) TCMK-1 cell survival was determined by CCK-8 assay. (**B**,**C**) Flow cytometry to detect TCMK-1 cell apoptosis and ROS levels. (**D**,**E**) RT-qPCR to detect the relative expression levels of renal *TFRC* and *PTGS2* mRNA. (**F**–**I**) ELISA results for IL-1β, IL-6, IL-10, and TNF-β in TCMK-1. Data are expressed as mean ± standard deviation (SD); ns: no significance; * *p* < 0.05; ** *p* < 0.01, *** *p* < 0.001; **** *p* < 0.0001.

Figure 3A,B shows that HR treatment inhibited cell survival and promoted cell death, as most significantly expressed at the ZT12 time point. Meanwhile, as shown in Figure 3C–E, markers associated with ferroptosis were altered after HR treatment, as observed through elevated ROS levels and increased *TFRC* and *PTGS2* mRNA levels, and these effects were most significant at the ZT12 time point. Fer-I significantly reversed the changes in the above parameters. As shown in Figure 3F–I, IL-1β and IL-6 were significantly elevated in the IR group compared with the results for the S group, and were more significant at the ZT12 time point. IL-1β and TNF-β were decreased in the IR+ Fer-I group. These results suggest that nocturnal HR treatment exacerbated oxidative stress and inflammatory injury in renal tubular epithelial cells, which may be associated with diurnal variability in ferroptosis.

### 3.4. Kidney Injury-Related Indexes and Gene Expression Changes in Knockdown of BMAL1 Gene

**Figure 4:**
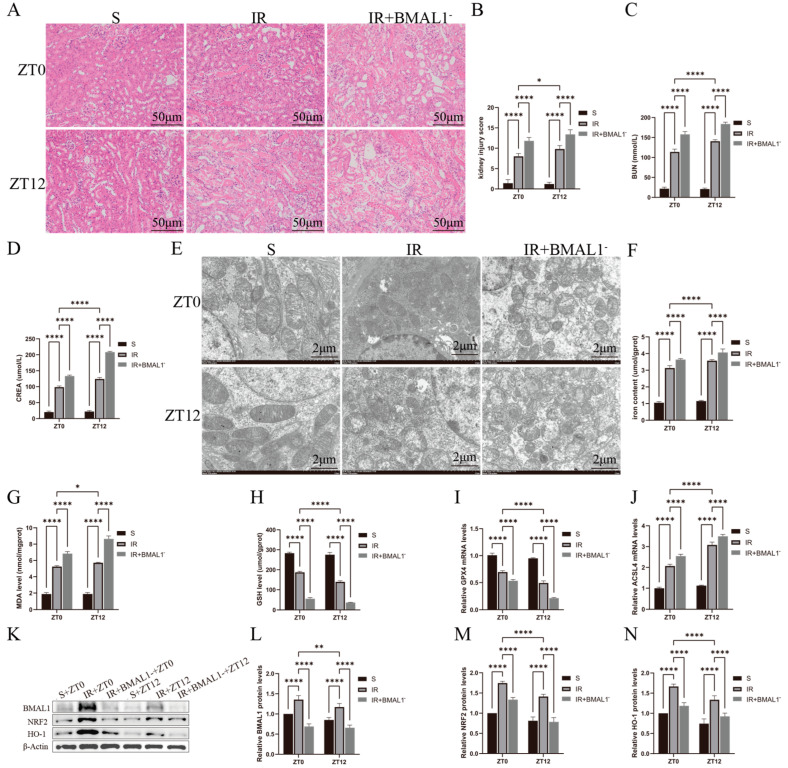
Knockdown of *BMAL1* gene renal injury-related indicators and gene expression changes. (**A**) Representative H&E staining images of kidney tissues used to assess IRI. Scale bar is 50 μm. (**B**) Renal H&E staining for renal injury score. (**C**,**D**) BUN and CREA levels in renal IR mice at ZT0 and ZT12 time points. (**E**) Representative images obtained by transmission electron microscopy. Scale bar represents 2 μm. (**F**–**H**) Total iron, malondialdehyde, and glutathione levels in renal tissues. (**I**,**J**) RT-qPCR detection of relative expression levels of renal *GPX4* and *ACSL4* mRNA. The mRNA levels in the sham-operated group were normalized to 1. (**K**) Relative expression levels of BMAL1, NRF2, and HO-1 proteins in renal tissues were detected by Western blotting. (**L**–**N**) Quantitative analysis of BMAL1, NRF2, and HO-1 proteins. Protein levels in the S group were normalized to 1. Data are expressed as mean ± standard deviation (SD); * *p* < 0.05; ** *p* < 0.01; **** *p* < 0.0001.

In Figure 4A, *BMAL1* knockdown exacerbated IR-induced renal tubular histologic changes and was more significant at ZT12. In Figure 4B, the histologic injury scores were higher in the IR+*BMAL1*- group than in the IR group and were more significant at ZT12. Similarly, in Figure 4C,D, BUN and CREA levels were elevated in the IR+*BMAL1* group compared with the IR group, especially at the ZT12 time point. In addition, transmission electron microscopy results, shown in Figure 4E, revealed that *BMAL1* knockdown exacerbated renal IR-induced mitochondrial structural disruption, and the results were more pronounced at ZT12. Total iron and MDA levels were higher in the IR+*BMAL1* group than in the IR group, and were more pronounced at the ZT12 node (Figure 4F,G). GSH levels and *GPX4* mRNA levels were significantly lower in the IR+*BMAL1* group than in the IR group and were more significant at ZT12 (Figure 4H,I). Figure 4J shows that the changes in *ACSL4* mRNA levels were the opposite of those for *GPX4*. Figure 4K shows the changes in BMAL1, NRF2, and HO-1 protein expression in kidney tissues under different treatments. Figure 4L–N shows that NRF2 protein expression in the IR+*BMAL1* group was lower than that in IR group and more significant at ZT12. In summary, *BMAL1* knockdown inhibited the rhythmic aggregation of NRF2 protein in the renal IRI model, leading to a significant elevation of nocturnal ferroptosis-related indices, suggesting that *BMAL1* may regulate the circadian variability of ferroptosis in renal IR by interfering with the *NRF2* pathway.

### 3.5. Changes in Ferroptosis Indicators and Circadian Gene Expression in the HR Model of Renal Tubular Epithelial Cells with Silencing of BMAL1 Gene

**Figure 5:**
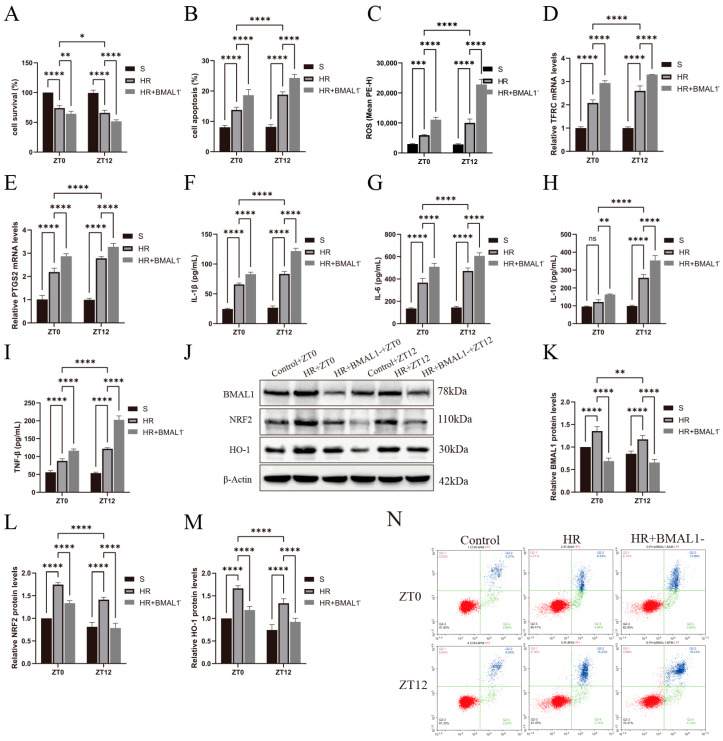
Changes in ferroptosis indicators and circadian gene expression in HR model of renal tubular epithelial cells with silenced *BMAL1* gene. (**A**) TCMK-1 cell survival was determined by CCK-8 assay. (**B**,**C**) Flow cytometry to detect TCMK-1 cell apoptosis and ROS levels. (**D**,**E**) RT-qPCR to detect the relative expression levels of renal *TFRC* and *PTGS2* mRNA. (**F**–**I**) ELISA results for IL-1β, IL-6, IL-10, and TNF-β in TCMK-1. (**J**) Western blotting results for relative expression levels of BMAL1, NRF2, and HO-1 proteins in TCMK-1. (**K**–**M**) Quantitative analysis of BMAL1, NRF2, and HO-1 proteins; group S protein levels were normalized to 1. (**N**) The four-quadrant histogram of Annexin-V-PI dual staining of flow cytometry apoptosis. Data are expressed as mean ± standard deviation (SD); ns: no significance; * *p* < 0.05; ** *p* < 0.01; *** *p* < 0.001; **** *p* < 0.0001.

Figure 5A,B shows that silencing the *BMAL1* gene aggravated cell death in the HR model and was more significant at the ZT12 time point. Meanwhile, as shown in Figure 5C–E, the silencing of the *BMAL1* gene aggravated cell ferroptosis, with elevated ROS levels and increased *TFRC* and *PTGS2* mRNA levels, especially at the ZT12 time point. As shown in Figure 5F–I, IL-1β, IL-6, IL-10, and TNF-β were significantly elevated in the HR+*BMAL1* group compared to the HR group, and more significantly at ZT12. Figure 5J shows the changes in BMAL1, NRF2, and HO-1 protein expression in the renal tubular epithelial cells under different treatments. Figure 5K–M showed that NRF2 protein expression was lower in the HR+*BMAL1* group than in the HR group and was more significant at ZT12. It was further confirmed in in vitro experiments that *BMAL1* was involved in the diurnal differential changes in ferroptosis in the HR model of renal tubular epithelial cells through NRF2 protein.

## 4. Discussion

In recent years, kidney transplantation protocols have been improved, with significantly higher short-term graft success rates and gradually improved long-term survival rates. However, the opportunities for further improvement are shrinking, and clinical diagnostic and treatment programs for kidney transplantation patients need to be continuously innovated and challenged [17]. During transplantation, transplanted kidneys inevitably experience IRI, which poses a significant challenge to maintaining the function and viability of the transplanted organ. It has been found that there is a significant difference in long-term graft survival when kidney transplantation is performed at different times of the day [18]. Meanwhile, the diurnal variability of renal injury in a mouse IR model has been confirmed, both in this paper and in our previous study [6], and this difference has also been found in an in vitro model of hypoxic reoxygenated renal tubular epithelial cells [19]. The above suggests that there may be diurnal variability in IR injury and its repair process experienced by transplanted kidneys, which may be of value in guiding future clinical protocols.

Ferroptosis is a form of cell death that is dependent upon iron and lipid peroxidation, involves dysregulation of iron–heme metabolism, and can be triggered by the stimulated generation of reactive oxygen species (ROS), which can lead to massive cell death [20]. IRI leads to elevated levels of oxidative stress in renal tissues, and ferroptosis leads to further elevation of ROS, exacerbating renal injury. The mechanism of ferroptosis in cardiovascular and other diseases has been gradually explored, and is expected to serve as a novel target for disease treatment [21,22,23]. Ferroptosis has been shown to correlate with pathways such as the circadian gene *BMAL1* and the *EBF3/ALOX15* axis. In this paper, we found that there were significant differences in iron content, MDA levels, and iron death-related factors, such as *GPX4* and *AXSL4*, at the ZT0 and ZT12 time points in the mouse renal IRI model, that Fer-I treatment suppressed the occurrence of ferroptosis, and that there were still differences between the ZT0 and ZT12 time points, indicating that there was a significant circadian variability of ferroptosis in the renal IRI model.

In this paper, both in vivo and in vitro experiments confirmed the correlation between circadian genes and ferroptosis. Silencing of the *BMAL1* gene caused a decrease in NRF2 protein, an increase in indicators of ferroptosis, as well as an aggravation of renal injury, whereas Fer-I was able to reverse the change, suggesting that *BMAL1* may be involved in the process of the ferroptosis of renal tubular epithelial cells in the renal IRI model through NRF2 protein. Studies have shown that *NRF2*, as an anti-oxidative stress coregulator, can inhibit ROS production, block cellular inflammatory responses, and influence cytokine production [24]. *BMAL1* regulates the expression of *NRF2* and its downstream anti-oxidative stress proteins through the *E-box* element binding to the PPAR promoter, while NRF2 regulates the expression of the downstream target genes of *BMAL1* via the regulation of Rev-Erbα (NR1D1) to inhibit *CLOCK/BMAL1* homeostasis [25]. Circadian oscillations in *BMAL1* expression also lead to periodic changes in NRF2 levels. Rhythmic *CLOCK* occupancy was also observed in the *E-box* region of the *NRF2* promoter [26]. When this *E-box* is mutated, binding is blocked [8], and *NRF2* expression is decreased during oxidative stress. Under quiescent conditions, NRF2 interacts with the protein Keap1 to maintain the low basal expression of NRF2-regulated genes. However, under oxidative stress conditions, NRF2 is released from Keap1 and translocated to the nucleus, activating intracellular gene expression and increasing cellular antioxidant capacity, thereby improving cell survival [27]. *BMAL1* and *Keap1* maintain daily variability in NRF2 levels under circadian rhythms and NRF2 activation during oxidative stress. *GPX4* is an important antioxidant enzyme, especially in the scavenging of lipid peroxides in cell membranes. NRF2, as an upstream regulator of *GPX4*, can upregulate the expression of *GPX4* through direct or indirect mechanisms, thus enhancing the resistance of cells to lipid peroxidation [28]. IRI disrupts the circadian gene *BMAL1*-mediated rhythmic recruitment of *NRF2* and impairs the protective effect of the *NRF2/ARE* pathway in nocturnal renal IRI, leading to the dysregulation of lipid peroxidation and iron metabolism and the development of massive production of ROS and ferroptosis, which exacerbates renal tissue injury.

*NRF2* is an important transcription factor. In ischemia–reperfusion injury, when cells are exposed to oxidative stress, *NRF2* is activated and translocated to the nucleus to activate ARE [6], which in turn upregulates the transcription and expression of downstream antioxidant enzymes like HO-1 to mitigate cellular damage caused by oxidative stress. Studies have shown that isoliquiritigenin(ISL) improves mitochondrial function and provides neuroprotection by activating the *NRF2* signaling pathway [29]. However, if ischemia extends for too long, and cell damage is aggravated, *NRF2* expression and activity may be inhibited, or negative feedback regulation may even occur, reducing *NRF2* activity. The effect of ischemia time on *NRF2* is influenced by a variety of factors, including the degree of ischemia, cell type, post-ischemic recovery, etc.

The *CLOCK-BMAL1* heterodimer directly or indirectly activates the transcription of various clock-controlled genes. The heterodimer also activates the transcription of several clock genes, including *Period (Per)1*, *Per2*, and the cryptochromes *CRY1* and *CRY2*. The PER and CRY proteins then translocate back to the nucleus and inhibit *CLOCK-BMAL1* activity, creating a negative feedback loop [30]. Therefore, it is reasonable to speculate that other circadian genes may also play a protective role in oxidative stress in renal IRI, similar to that of the *BMAL1/NRF2* pathway.

Based on our experimental results, circadian modulation of IRI can be applied in clinical medicine to align the timing of treatment with the body’s natural rhythm. Certain levels of IRI can have a decisive impact on many kidney injuries, so understanding the peak times when the kidney is more vulnerable or more resistant to injury could guide more effective treatment strategies. For example, if the body is more resistant to renal IRI at certain times of the day, then doctors can schedule surgery or protective interventions during those times to minimize injury. Currently, chronotherapies based on circadian rhythms for the treatment of disease are now emerging. Chronotherapy aims to promote health by improving the body’s synchronization with natural rhythms. With respect to the advancement of research on chronotherapy in recent years [31,32], more research is required to optimize the treatment scheme based on the specific characteristics of circadian rhythms for patients following renal transplantation.

In summary, the circadian gene *BMAL1* affects the circadian variability of ferroptosis in renal IRI through the regulation of *NRF2* and its downstream pathway. The experimental application of ferroptosis inhibitors significantly ameliorated renal injury and provided preventive and diagnostic possibilities, as well as relevant targets for clinical application, which are expected to reduce the generation of oxidative stress and decrease renal injury. The study focused on the regulation of *NRF2* by *BMAL1* and neglected other factors that can affect ferroptosis in renal IR injury. Additionally, the animal experiments lacked the study of a mouse kidney transplantation model, and its difference in the mechanism of action from that of the IRI model needs to be explored in depth in the future.

## Data Availability

Core data have been provided in the main text. The data can be made available upon reasonable request.

## Abbreviations

The following abbreviations are used in this manuscript:

IRI	Ischemia reperfusion injury
HR	Hypoxia–reoxygenation
ROS	Reactive oxygen species
Fer-I	Ferroptosis inhibitor
MDA	Malondialdehyde
